# A rare case of unilateral Cogan’s anterior internuclear ophthalmoplegia, upgaze palsy and ataxia caused by dorsal tegmentum lesion at pontomesencephalic junction

**DOI:** 10.1186/s12886-021-01868-9

**Published:** 2021-02-25

**Authors:** Yong Zheng Wai, Qi Xiong Ng, Tsu Hong Lim, Lik Thai Lim

**Affiliations:** 1 Queen Elizabeth Hospital, Sabah, Malaysia; 2 Universiti Malaysia Sarawak (UNIMAS), Kota Samarahan, Sarawak, Malaysia

**Keywords:** Cogan’s anterior internuclear ophthalmoplegia, Pontomesencephalic junction

## Abstract

**Background:**

Cogan’s anterior internuclear ophthalmoplegia (INO) is characterized by INO with inability to converge and commonly thought to be due to rostral midbrain lesion. A lesion outside midbrain that causes unilateral Cogan’s anterior INO combined with upgaze palsy and ataxia are rarely described.

**Case presentation:**

A 67-year old male presented with left Cogan’s anterior internuclear ophthalmoplegia (INO), left appendicular ataxia and bilateral upgaze palsy. A Magnetic Resonance Imaging (MRI) and Magnetic Resonance Angiography (MRA) brain showed a left dorsal tegmental infarct at the level of pontomesencephalic junction.

**Conclusions:**

This case highlights the clinical importance of Cogan’s anterior INO in combination with upgaze palsy and ataxia, and report possible site of lesion in patients with such constellation. Clinicians should consider looking for cerebellar signs in cases of Cogan’s anterior INO, apart from just considering localizing the lesion at the midbrain.

## Background

Internuclear ophthalmoplegia (INO) is a discrete localizing sign which narrows down the lesion involving medial longitudinal fasciculus (MLF) anywhere at the paramedian tegmentum from caudal pons to midbrain [1]. The MLF relays the contralateral abducens nucleus to ipsilateral medial rectus subnucleus of the oculomotor nuclear complex [2]. Cogan further classified INO into anterior and posterior variety. In which anterior INO shows convergence impairment, whereas posterior INO exhibits intact convergence [3]. He proposed that the presence of anterior INO helps to further localize the lesion over the most rostral portion of MLF conducting the impulses from the pretectal region to the 3rd nerve nucleus in midbrain, whereas posterior INO indicates lesions at the level of the 4th ventricles in pons [4].

INO combined with ataxia has rarely been described. Most literature reported that the lesions were located in the midbrain [1, 5, 6]. There is a paucity of cases in literatures reporting on Cogan’s anterior INO with ataxia. To the authors’ best knowledge, there are only two case reports on Cogan’s anterior INO, both of which do not have ataxia [7, 8].

This case demonstrates unilateral Cogan’s anterior INO with ipsilateral limb ataxia in which the lesion falls outside the midbrain, located in the pontomesencephalic junction. This case highlights the importance of considering pontomesencephalic junction as one of the possibilities in a case with such constellation.

## Case presentation

### History

A 67-year-old gentleman with undiagnosed dyslipidemia and hypertension presented with difficulty in walking and painless diplopia for 1 week. He noticed the symptoms after waking up from sleep.

He denied any head trauma, headache, nausea, or vomiting. There were no associated sensory, motor, speech, and vertigo symptoms. There was no family history of cerebral vascular accident or ischaemic heart disease. His ocular history was insignificant, with no previous ocular surgery. He had never undergone any medical check-up.

### Physical examination

The vital sign showed elevated blood pressure (175/89 mmHg) with normal random blood sugar (6.7 mmol/L). Visual acuity with a pinhole was 6/10 OD (right eye) and 6/15 OS (left eye). Both pupils were round and equal in size (3mm) with a sluggish direct light reflex. The swinging light test showed no relative afferent pupillary defect.

**Fig. 1:**
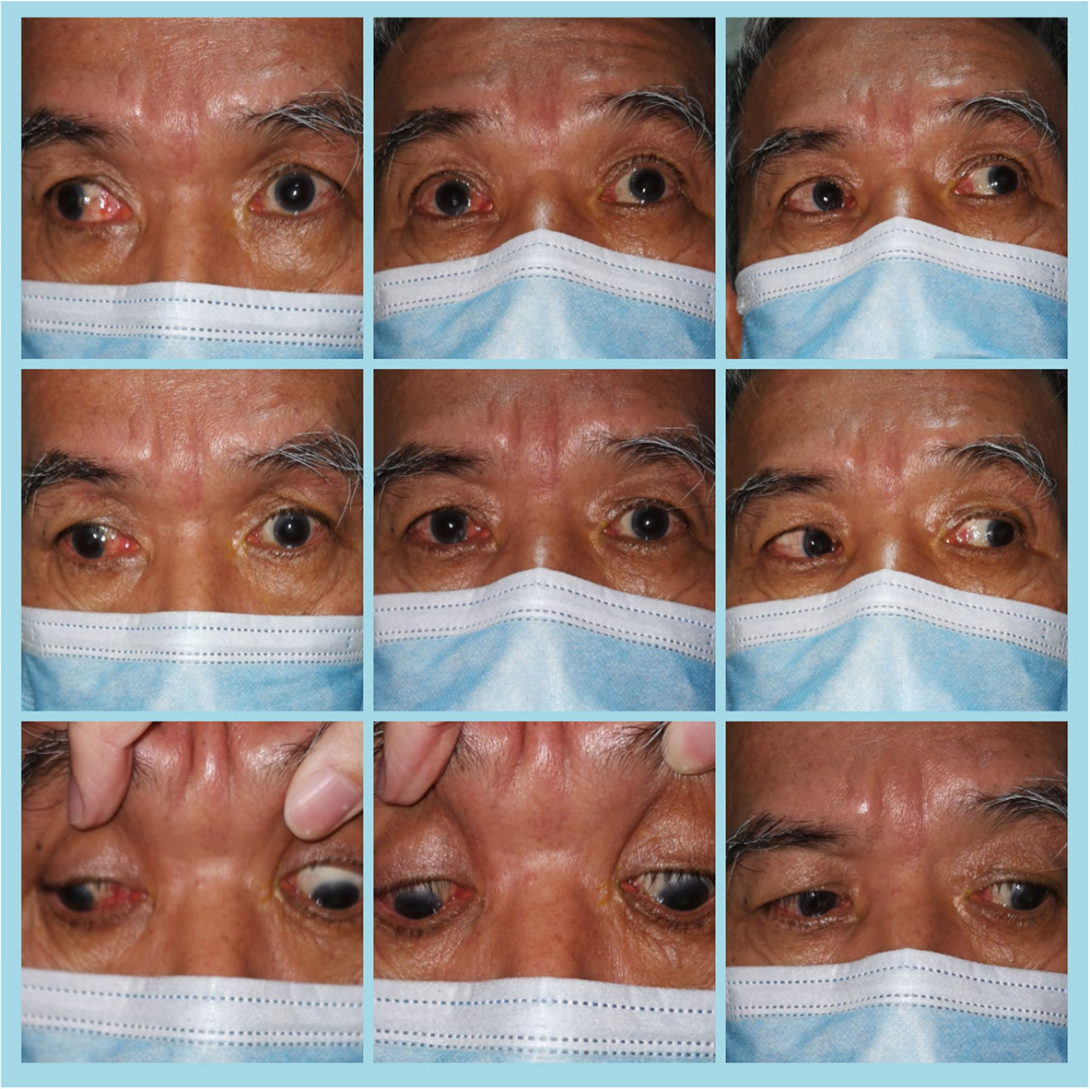
In the 9 diagnostic positions of gaze, left eye unable to adduct beyond the midline with right eye abduction nystagmus and marked upgaze limitation

On primary gaze, eyes were central on Hirschberg light reflex test. There was no ptosis, proptosis, or abnormal head posture. Assessment of extraocular muscle motility revealed a horizontal abducting nystagmus in the right eye in right gaze and left eye was unable to adduct beyond the midline on dextroversion. Besides that, there was a limitation in elevation in both eyes, with normal depression.(Fig. 1) He experienced diplopia only in dextroversion, dextroelevation and dextrodepression. He was unable to converge, hence light-near dissociation could not be elicited. Slit-lamp examination showed cataract in both eyes. Otherwise, his anterior and posterior segments were normal in both eyes.

Moving on to neurological examination, the patient was alert and conscious with a full Glasgow Coma Scale. His speech was clear, coherent, and relevant. Besides the abnormal ocular motility, other cranial nerves were all intact. The sensory and motor function of all 4 limbs were full and of normal tone. Proprioception and plantar reflex were unremarkable. However, he had bilateral appendicular ataxia in both upper and lower limbs worse in the left side. He was frequently falling towards the left side on tandem gait assessment.

## Investigations

**Fig. 2:**
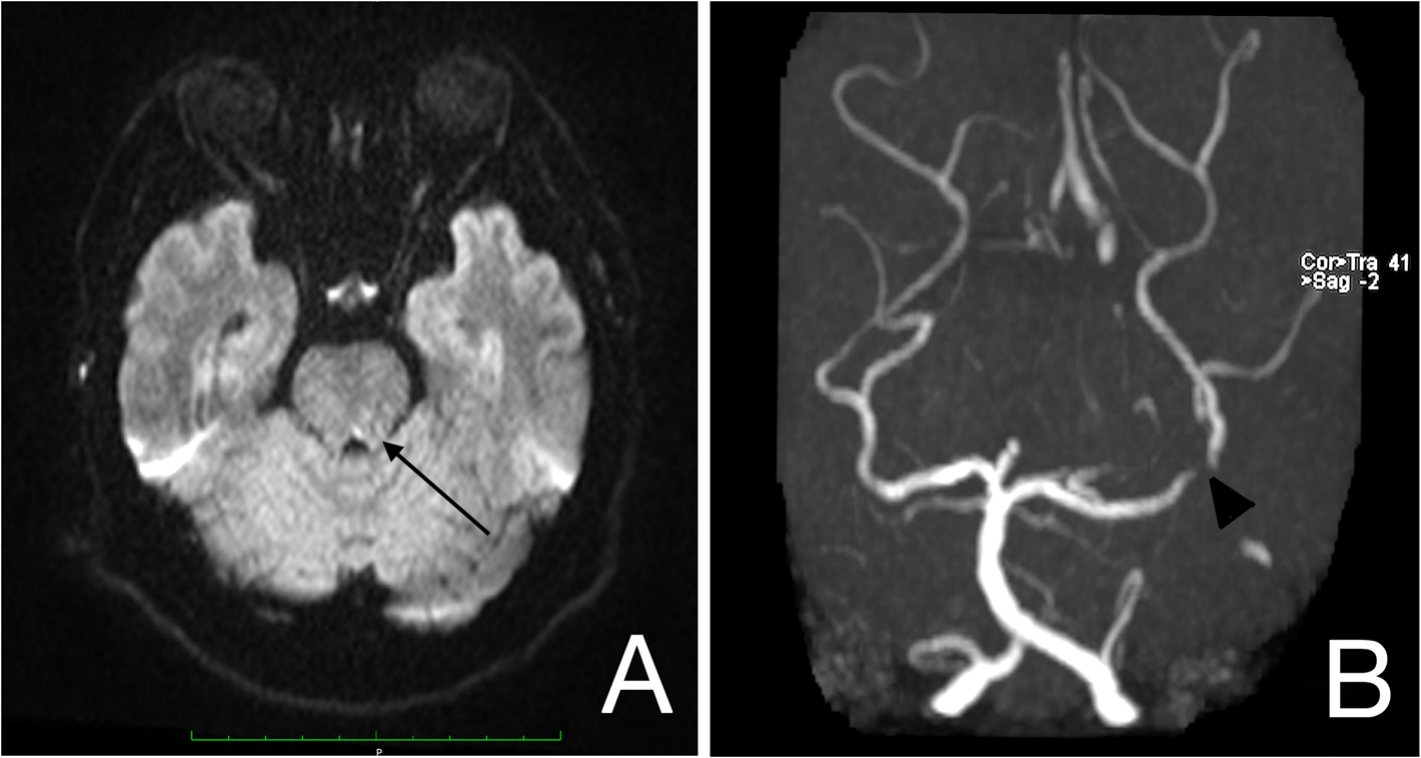
MRI brain of axial diffusion-weighted imaging sequences showed a hyperintense lesion at the left dorsal tegmentum of pontomesencephalic junction (**a**). MRA revealed narrowing at the P2 segment of the left posterior cerebral artery (**b**)

A brain Magnetic Resonance Imaging (MRI) and Magnetic Resonance Angiogram (MRA) with gadolinium showed hyperintense lesion (lacunar infarct) over left dorsal pontomesencephalic junction associated with atherosclerotic disease with stenosis at P2 Segment of left posterior cerebral artery. **(**Fig. 2**)** The cerebellum was normal. Humphrey visual field showed no visual field abnormalities.

Fasting serum lipid showed a total cholesterol level of 7.1 mmol/L with LDL of 5.2 mmol/L.

## Management

After reviewing the investigation and correlating with the clinical signs, the patient was diagnosed infarct of the left dorsal tegmentum of pontomesencephalic junction. During the admission, his ataxia was improving and able to ambulate without any support upon discharge. He was given oral Simvastatin, antihypertensive agent and aspirin. Patient was advice to modify his lifestyle (cut down smoking and alcohol intake). He was given an appointment with neurology and ophthalmology clinic before discharged.

After 1 month, his diplopia and ataxia had significant improved. The extraocular movements were almost full with minimal cerebellar sign seen.

## Discussion and conclusion

**Fig. 3:**
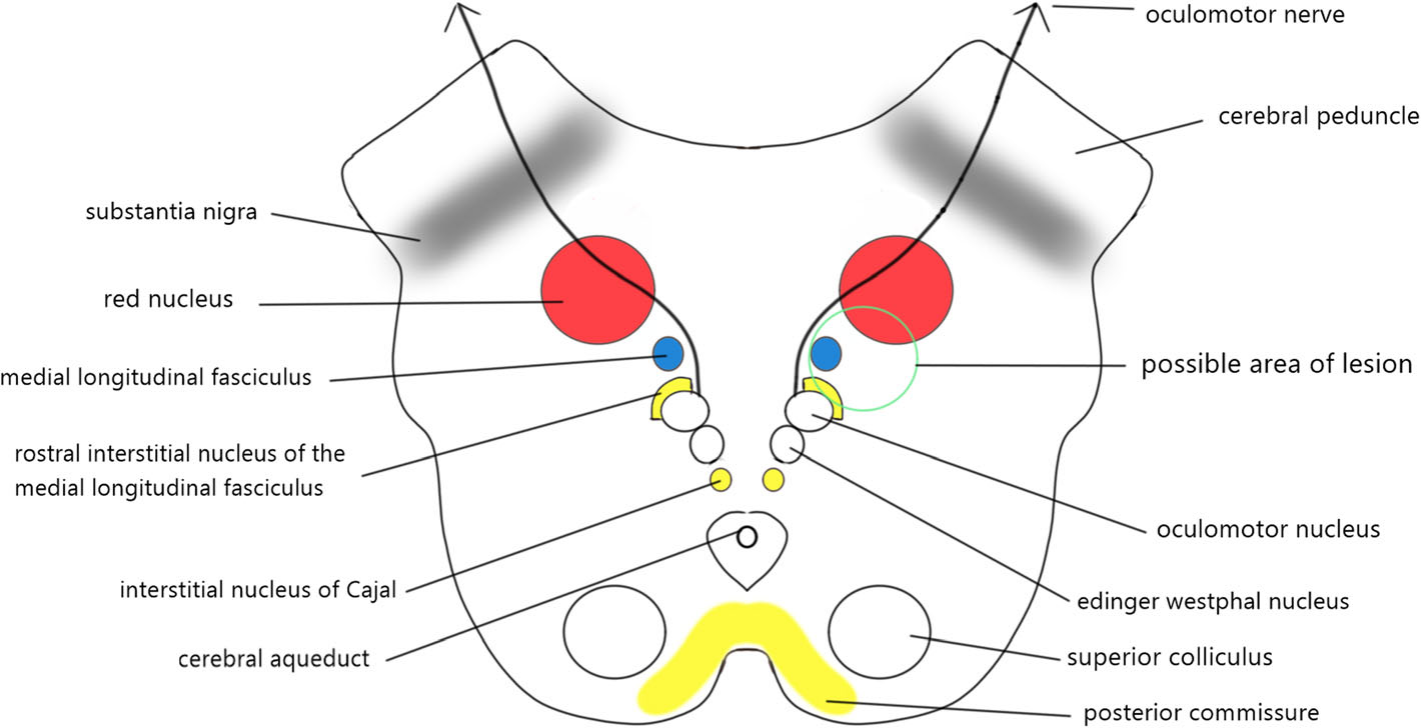
Schematic diagram of the rostral midbrain at the level of the superior colliculus. If the lesion involved red nucleus, medial longitudinal fasciculus (MLF), and rostral interstitial medial longitudinal fasciculus (riMLF), the patient might have similar signs as seen in our patient

This patient presented with three main clinical signs which include unilateral Cogan’s anterior INO, upgaze palsy, and appendicular ataxia. A constellation of these 3 signs are rare and made clinicians localized the lesion at the level of the midbrain [3, 5, 6, 9, 10]. Rostral midbrain contains important structures for vertical gaze, which includes rostral interstitial medial longitudinal fasciculus (riMLF), interstitial nuclear of Cajal (INC) and posterior commissure (PC) [11]. Injury over any one of these structures leads to vertical gaze abnormalities [10]. Red nucleus located anterior to the 3rd nerve nucleus and any lesion over the red nucleus can cause contralateral ataxia, tremors and choreiform activity due to rubrospinal tract that supply the contralateral body [12]. MLF lies between the red nucleus and 3rd nerve nuclei [12]. A lesion at the paramedian tegmentum of rostral midbrain (superior colliculus level) which involves red nucleus, MLF, and riMLF can cause similar signs except the ataxia will be contralateral [11, 12] **(**Fig. 3**)**.

However, the MRI brain of this patient showed there was dorsal tegmentum lesion at the pontomesencephalic junction instead of the paramedian midbrain. In order to understand this better, we will need to localize lesions for each of the 3 signs to pinpoint the exact location of the lesion.

Cogan’s anterior INO is defined as INO superimposed with inability to converge [3]. Initially, it was thought that this sign can be helpful to localize the lesion more rostral in the midbrain, where MLF connects with the subnucleus of 3rd nerve nucleus [4]. This concept is generally accepted by most clinicians [13]. However, further experiments and studies showed that this is unreliable due to inappropriate vergence signal carried by MLF [2, 14]. Thus, absence of convergence in INO has not much implication in localization of the lesion, but present of convergence is useful to rule out pseudo-INO [15]. Our case is in-line with other studies, as a lesion in pontomesencephalic junction was able to impair convergence and produce the Cogan’s anterior INO.

**Fig. 4:**
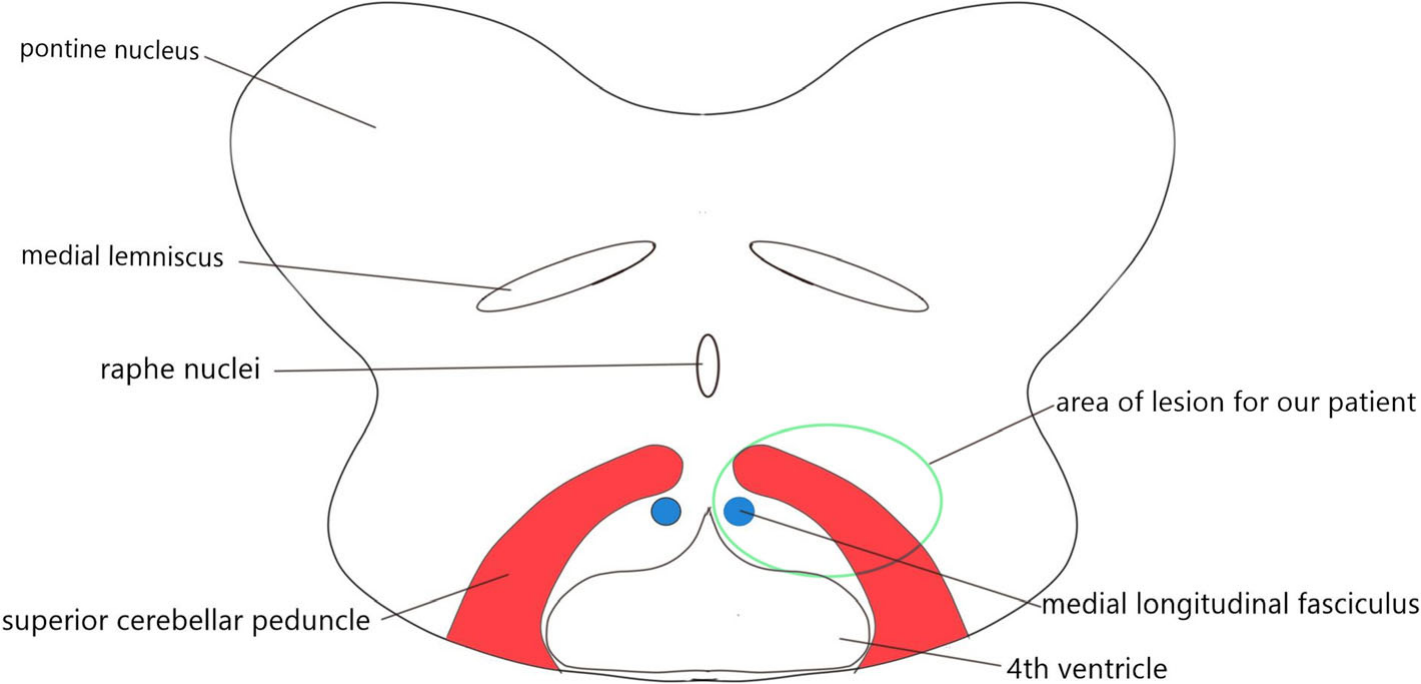
Schematic diagram of the pontomesencephalic junction. Our patient has left dorsal tegmentum infarct at pontomesencephalic junction, which involved medial longitudinal fasciculus (MLF) and superior cerebellar peduncle

Left Cogan’s anterior INO plus appendicular ataxia are rare. This combination narrowed the location to the left dorsomedial tegmentum of pontomesencephalic junction, left paramedian tegmentum of the caudal midbrain, and left paramedian tegmentum of rostral midbrain. In this case, MLF origin from the right paramedian pontine reticular formation (PPRF) at the level of abducens nerve nuclei in the caudal pons [15]. The MLF decussate to the left at the same level and ascend along the dorsomedial tegmentum of pons. At pontomesencephalic junction, superior cerebellar peduncle enters laterally to the brainstem which connects the dentate nucleus to the red nucleus and thalamus. An infarct circumscribed to the superior cerebellar peduncle can result in ipsilesional limb ataxia [16]. MLF is located dorsomedially to the superior cerebellar peduncle (Fig. 4)[17, 18]. MLF transmits signal for vertical movement and graviception from contralateral vestibular nuclei to Internuclear of Cajal (INC) in upper midbrain [11]. Hence, a lesion at the left dorsal tegmentum of pontomesencephalic junction which affects the left MLF and left superior cerebellar peduncle can present with left INO, upgaze palsy with left limb ataxia [18].

**Fig. 5:**
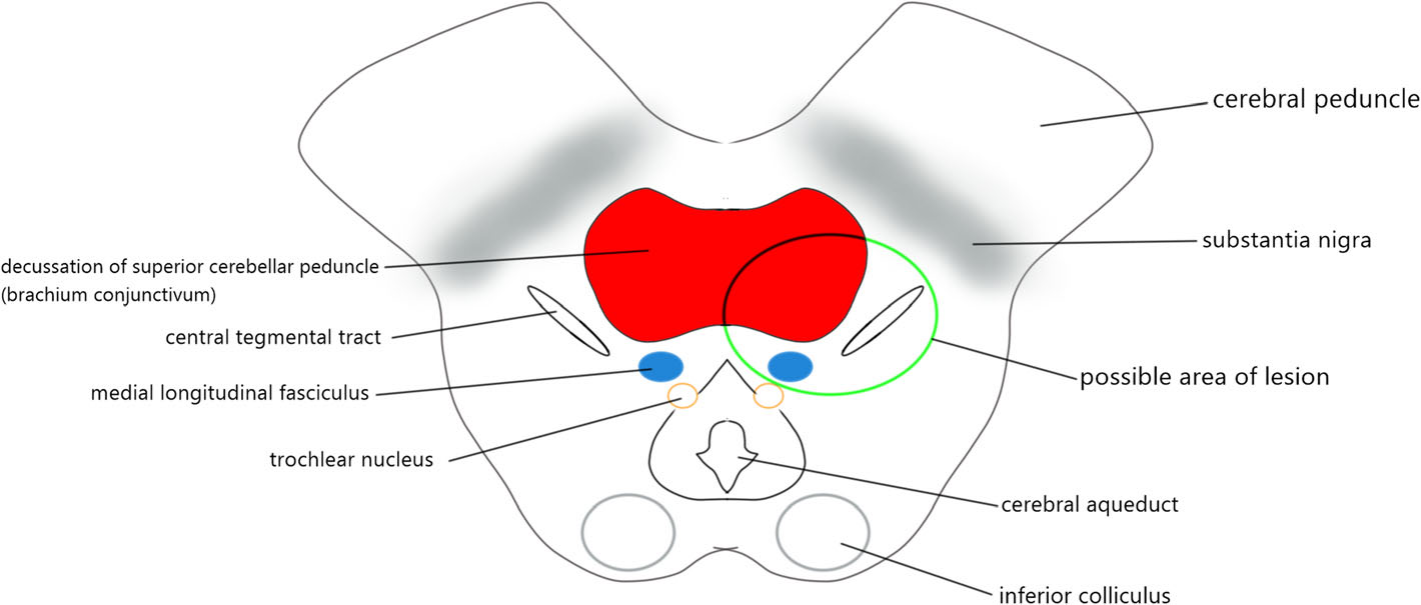
Schematic diagram of caudal midbrain at the level of the inferior colliculus. Involvement of medial longitudinal fasciculus (MLF) and decussation of superior cerebellar peduncle could present with similar complaints as our patient

The superior cerebellar peduncle (also known as brachium conjunctivum) ascends to the caudal pons and decussates over the midline at the level of inferior colliculus before their entrance to the red nucleus, this decussation point is called as Wernekinck commissure [12, 19]. Wernekinck commissure is located ventral to the aqueduct. Lesion above this level causes contralateral cerebellar sign, while lesion below this level manifest ipsilateral cerebellar sign [12]. MLF is located dorsal to the decussation of brachium conjunctivum (Fig. 5). Few cases reported paramedian tegmentum lesion over caudal midbrain result in INO with ataxia, also known as Wernekinck commissure syndrome [19–21]. However, none of them commend convergence ability. Besides this, the superior cerebellar peduncle contributes to the vertical smooth pursuit and eye-head tracking as it transmits the gaze-velocity signal from the dorsal portion of the y-group to 3rd cranial nerve nucleus [11]. Thus, a lesion over this area can present similar signs as seen in our patient (Fig. 5).

Cogan’s INO, upgaze palsy with ataxia can be due to lesion located at the 3 levels as mentioned above (dorsomedial tegmentum of pontomesencephalic junction, paramedian tegmentum of caudal midbrain, and paramedian tegmentum of rostral midbrain). Among patients with INO, 40 % of INO with ataxia patients unable to converge, compared to isolated INO only 10 % had convergence impairment [18]. Cogan’s anterior INO will be a useful sign for clinicians to emphasize on the cerebellar examination, rather than localization of the lesion (Table 1).

**Table 1:** Summary of the clinical signs and location of the lesion

Location of the lesion	Cogan’s Anterior INO	Cerebellar sign
Paramedian Tegmentum of Rostral Midbrain (Fig. 3)	Present	Contralateral cerebellar ataxia
Paramedian Tegmentum of Caudal Midbrain (Fig. 5)	Present	Bilateral cerebellar ataxia
Dorsomedial Tegmentum of Pontomesencephalic Junction (Fig. 4)	Present	Ipsilateral cerebellar ataxia

INO with vertical gaze palsy is rarely seen with lesions in the upper midbrain, and to our best knowledge, there are only 2 cases reported [22]. In cases with lesions at the level of pontomesencephalic junction, vertical palsy is present in 23 % of INO with ataxia and none of the isolated INO patients has vertical gaze palsy [18]. Hence, vertical gaze palsy with INO can hint clinicians to focus over the pontomesencephalic junction and check for cerebellar involvement. Most of the cases of INO with ataxia are caused by vascular infarct [1, 18, 20, 22]. In our case, he experienced all the symptoms after waking up in the morning. We therefore hypothesized nocturnal hypotension could be the aetiology of cerebral vascular infarct. Prognosis of Cogan’s anterior INO secondary to vascular infarct is good, symptoms usually improve with time.

## Conclusions

This is a rare case report with a constellation of unilateral Cogan’s anterior INO, upgaze palsy with ataxia due to a dorsal tegmental lesion at pontomesencephalic junction. Clinicians should consider looking for cerebellar sign in cases of Cogan’s anterior INO, apart from just considering localizing the lesion over the midbrain.

INO with vertical gaze palsy should alert clinicians to look for lesions near pontomesencephalic junction and cerebellar involvement. INO with ataxia is mostly due to vascular infarct.

## Data Availability

All data and materials gathered during this study are included in this study.

## Abbreviations

INO: Internuclear ophthalmoplegia
MLF: Medial longitudinal fasciculus
riMLF: Rostral interstitial medial longitudinal fasciculus
INC: Interstitial nuclear of Cajal
MRI: Magnetic resonance imaging
MRA: Magnetic resonance angiography

## References

[1] Kim JS (2004). Internuclear ophthalmoplegia as an isolated or predominant symptom of brainstem infarction. Neurology.

[2] Leigh RJ, Zee DS. The Neurology of Eye Movements [Internet]. Oxford, UK: Oxford University Press; 2015. Available from: https://oxfordmedicine.com/view/10.1093/med/9780199969289.001.0001/med-9780199969289.

[3] Cogan DG. Internuclear ophthalmoplegia, typical and atypical. Arch Ophthalmol (Chicago, Ill 1960). 1970;84(5):583–9. 10.1001/archopht.1970.00990040585005 5478883

[4] Kupfer C, Cogan DG. Unilateral internuclear ophthalmoplegia. A clinicopathological case report. Arch Ophthalmol (Chicago, Ill 1960). 1966;75(4):484–9. 10.1001/archopht.1966.00970050486008 5905203

[5] Okuda B, Tachibana H, Sugita M, Maeda Y. Bilateral internuclear ophthalmoplegia, ataxia, and tremor from a midbrain infarction. Vol. 24, Stroke. United States; 1993. p. 481–2. 10.1161/01.str.24.3.481 8446990

[6] Bogousslavsky J, Maeder P, Regli F, Meuli R. Pure midbrain infarction. Neurology [Internet]. 1994 Nov 1;44(11):2032 LP – 2032. Available from: http://n.neurology.org/content/44/11/2032.abstract. 10.1212/wnl.44.11.2032 7969955

[7] Gelaw Y. Cogan’s anterior internuclear ophthalmoplegia in young Ethiopian: A case report and review of literature. Alexandria J Med [Internet]. 2014;50(4):373–6. Available from: 10.1016/j.ajme.2014.05.006.

[8] Khan SA, Brooks M, Crompton DE (2015). Large basilar tip aneurysm causing anterior internuclear ophthalmoplegia. Pract Neurol.

[9] Pollak L, Zehavi-Dorin T, Eyal A, Milo R, Huna-Baron R (2017). Parinaud syndrome: Any clinicoradiological correlation?. Acta Neurol Scand.

[10] Yang Y, Qidwai U, Burton BJL, Canepa C (2020). Bilateral, vertical supranuclear gaze palsy following unilateral midbrain infarct. BMJ Case Rep.

[11] Bhidayasiri R, Plant GT, Leigh RJ (2000). A hypothetical scheme for the brainstem control of vertical gaze. Neurology.

[12] Kurkcuoglu A. Mesencephalon. Midbrain. Hum Anat - Rev Med Adv. 2017.

[13] Haider AS (2016). Unilateral internuclear ophthalmoplegia, strabismus and transient torsional nystagmus in focal pontine infarction. BMJ Case Rep.

[14] Gamlin PDR, Gnadt JW, Mays LE (1989). Lidocaine-induced unilateral internuclear ophthalmoplegia: Effects of convergence and conjugate eye movements. J Neurophysiol.

[15] Virgo JD, Plant GT (2017). Internuclear ophthalmoplegia. Pract Neurol.

[16] Lee SU, Bae HJ, Kim JS. Ipsilesional limb ataxia and truncal ipsipulsion in isolated infarction of the superior cerebellar peduncle. J Neurol Sci [Internet]. 2015;349(1–2):251–3. Available from: 10.1016/j.jns.2015.01.006. 10.1016/j.jns.2015.01.006 25592415

[17] Matsushima K, Yagmurlu K, Kohno M, Rhoton AL (2016). Anatomy and approaches along the cerebellar-brainstem fissures. J Neurosurg.

[18] Lee SU, Kim HJ, Park JJ, Kim JS (2016). Internuclear ophthalmoplegia plus ataxia indicates a dorsomedial tegmental lesion at the pontomesencephalic junction. J Neurol.

[19] Liu H, Qiao L, He Z (2012). Wernekink commissure syndrome: A rare midbrain syndrome. Neurol Sci.

[20] Krespi Y, Aykutlu E, Çoban O, Tunçay R, Bahar S (2001). Internuclear ophthalmoplegia and cerebellar ataxia: Report of one case. Cerebrovasc Dis.

[21] Sheetal S, Byju P (2016). Wernekink commissure syndrome. Neurol India.

[22] Zhang Y, Wang L, He M. Isolated INO as a presentation of midbrain paramedian area lacunar infarction in patients with diabetes. J Clin Neurosci [Internet]. 2017;45:261–4. Available from: 10.1016/j.jocn.2017.08.005. 10.1016/j.jocn.2017.08.005 28869134

